# Recent advances in noncanonical inhibition mechanisms of anti‐CRISPR proteins

**DOI:** 10.1002/mlf2.70077

**Published:** 2026-04-10

**Authors:** Lingguang Yang, Rongjun Luo, Wei Zhou, Peipei Yin, Yue Feng, Yi Zhang

**Affiliations:** ^1^ Yichun University Center for Innovative Technologies in Gene‐Based Precision Therapeutics, School of Pharmacy Yichun University Yichun China; ^2^ Bloomage Biotechnology Co. Ltd. Jinan China; ^3^ State Key Laboratory of Green Biomanufacturing, College of Life Science and Technology Beijing University of Chemical Technology Beijing China

**Keywords:** anti‐CRISPR, CRISPR‐Cas, noncanonical inhibition, phage–host arms race, structural mechanism

## Abstract

The CRISPR‐Cas system constitutes an adaptive immune mechanism in prokaryotes that defends against mobile genetic elements. Within the perpetual co‐evolutionary arms race between bacteria and their viral predators, bacteriophages encode anti‐CRISPR (Acr) proteins that use sophisticated molecular strategies to sabotage CRISPR‐Cas function. While canonical Acr proteins rely on steric blockade of Cas effectors, recent discoveries reveal unprecedented noncanonical mechanisms spanning CRISPR immunity stages. This review synthesizes recent mechanistic advances in this field since 2023, highlighting the expansion of noncanonical inhibition mechanisms beyond type I to include types II, V, and VI, as well as novel Acr interventions targeting multiple functional stages, such as spacer acquisition, translation‐coupled inhibition, complex assembly/disassembly, and R‐loop DNA binding. Structural insights demonstrate how Acr proteins achieve substoichiometric inhibition via conformational hijacking, catalytic repurposing, and molecular mimicry. Forged by the intense selective pressure of the phage–host conflict, these molecular innovations represent both remarkable evolutionary adaptations and versatile precision tools. They enable spatiotemporal control of CRISPR technologies, from engineered off‐switches to diagnostic reset mechanisms, while posing critical challenges for therapeutic safety and microbiome management.

## INTRODUCTION

The relentless molecular arms race between prokaryotes and their viral predators has driven the evolution of sophisticated immune defenses and equally sophisticated countermeasures^1^. Among these, CRISPR‐Cas systems represent the only known adaptive immune machinery in prokaryotes, utilizing CRISPR RNA (crRNA)‐guided surveillance complexes to recognize and cleave invasive nucleic acids^2^. Having been repurposed as transformative genome‐editing platforms, these systems now revolutionize therapeutics, diagnostics, and synthetic biology^3^. In response, phages and other mobile genetic elements (MGEs) have evolved anti‐CRISPR (Acr) proteins as potent suppressors to neutralize CRISPR‐Cas immunity^4^. Furthermore, Acr proteins serve as precision regulators in applied settings, offering solutions to persistent challenges such as off‐target effects and the need for spatiotemporal control over editing^5^, ^6^. Since the landmark discovery of the first Acr proteins targeting type I–F systems in 2013, a growing number of distinct Acr families have been identified across CRISPR types I–VI, revealing remarkable mechanistic diversity^7^.

Early structural and biochemical studies established canonical inhibition strategies used by Acr proteins as steric obstruction of target DNA binding to CRISPR surveillance complexes such as Cascade, prevention of Cas nuclease recruitment, or direct inhibition of nuclease activity^8^. These mechanisms typically involve high‐affinity, stoichiometric interactions between Acr proteins and their matched Cas effectors, acting as molecular “masks” or competitive inhibitors. This steric occlusion defines the canonical inhibition paradigm, which is characterized by high‐affinity binding that physically blocks functional sites on Cas effectors, such as DNA‐binding pockets or nuclease domains^9^. In contrast, noncanonical strategies encompass mechanisms that diverge from simple obstruction. These often involve enzymatic activity to irreversibly inactivate Cas components or their nucleic acid guides, allosteric manipulation of Cas protein conformations, interference with complex assembly or stability, or substoichiometric activities that achieve potent inhibition without saturating the target^9^. The defining feature of noncanonical Acr is their ability to suppress CRISPR immunity through means other than direct, stable steric blockage.

Building on this definition, recent advances have unveiled a striking expansion of these noncanonical strategies that transcend simple blockage, reflecting the escalating sophistication of the phage–host arms race. These diverse approaches often operate substoichiometrically or exploit transient catalytic activities, thereby enhancing their potency in evading host defenses. Indeed, recent studies since 2023 have expanded these atypical mechanisms beyond type I to include types II, V, and VI, and revealed their action at novel functional stages such as spacer acquisition and translation‐coupled inhibition, as well as complex assembly/disassembly. The discovery of such diverse and potent mechanisms underscores the intense selective pressure within this evolutionary conflict. Critically, such mechanisms often operate substoichiometrically or exploit transient catalytic activities, enhancing their potency in evading host immunity.

To date, over 120 Acr families have been identified, spanning novel subtypes (e.g., AcrIA, AcrIB, and AcrVIB), underscoring accelerated discovery and mechanistic diversification^7^. This review focuses on the emergent frontier of noncanonical Acr inhibition mechanisms, emphasizing insights gleaned from high‐resolution structural biology and complementary functional studies since our last review in 2023^9^. These breakthroughs illuminate the escalating complexity of phage–prokaryote conflicts while providing transformative tools for biotechnology. Engineered Acr proteins exploiting noncanonical mechanisms enable enhanced spatiotemporal control over CRISPR‐based genome editing, diagnostics, and therapeutics. By synthesizing recent structural and mechanistic revelations, this review aims to catalyze further exploration of Acr diversity and inspire innovative applications in genome engineering and synthetic biology.

## CRISPR‐CAS SYSTEMS: MOLECULAR DEFENDERS AND GENOME EDITORS

**Figure 1:**
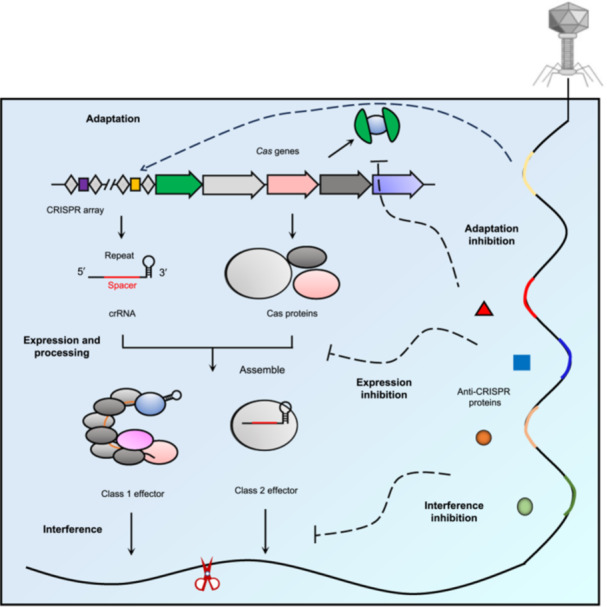
The CRISPR‐Cas adaptive immune system and its sabotage by Anti‐CRISPR (Acr) proteins. This schematic illustrates the three stages of CRISPR‐Cas adaptive immunity, including adaptation, expression and processing, and interference, and the diverse inhibition mechanisms used by bacteriophage‐encoded Acr proteins to neutralize each stage.

The discovery of CRISPR sequences began serendipitously in 1987 when Ishino's team observed unusual repetitive elements flanking the *iap* gene in *Escherichia coli*, though their biological function remained elusive^10^. Subsequent genomic analyses revealed conserved architectures across diverse prokaryotes, culminating in 2002, when Jansen et al. formally defined these as Clustered Regularly Interspaced Short Palindromic Repeats (CRISPR) while identifying conserved cas (CRISPR‐associated) genes (*Cas1*–*Cas4*)^11^. The immunological function was conclusively demonstrated in 2007, when Horvath's group showed that *Streptococcus thermophilus* could integrate phage‐derived spacers into CRISPR arrays, conferring sequence‐specific resistance to viral reinfection^12^. As an adaptive immune system, CRISPR‐Cas protects prokaryotes from MGEs through three evolutionarily conserved phases: adaptation, in which Cas1–Cas2 complexes recognize and integrate foreign DNA fragments (protospacers) into the host CRISPR array; expression and processing, where the array is transcribed into precursor CRISPR RNAs (pre‐crRNAs) that are processed into mature guide RNAs (crRNAs); and interference, where crRNA‐loaded effector complexes surveil and cleave complementary invasive nucleic acids bearing protospacer adjacent motifs (PAMs) (Figure 1)^13^, ^14^.

**Figure 2:**
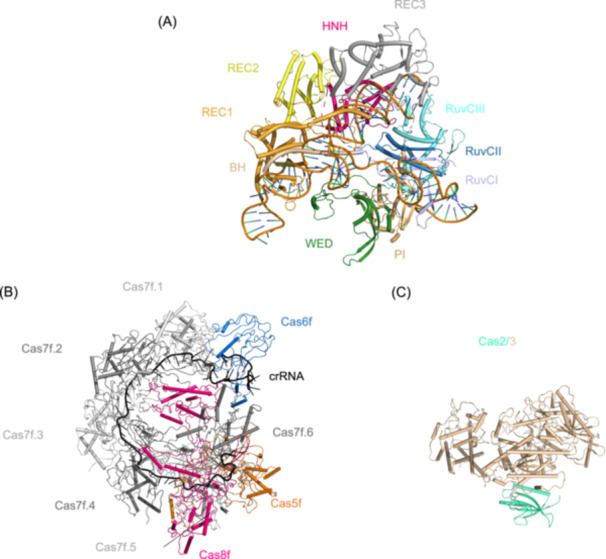
Representative structures of Class 1 and Class 2 CRISPR‐Cas effectors. (A) Structure of the single effector protein *Streptococcus pyogenes* Cas9 (SpyCas9) from the type II‐A system (PDB ID: 4OO8), showcasing the architecture common to Class 2 nucleases. (B) Architecture of the multi‐subunit type I‐F Cascade surveillance complex (PDB ID: 6B45), which performs crRNA‐guided DNA target recognition in Class 1 systems. (C) Structure of the Cas2/3 nuclease fusion protein (PDB ID: 5B7I), which is recruited by Cascade to cleave target DNA in the type I‐F system. BH, bridge helix; HNH, histidine‐asparagine‐histidine endonuclease domain; PI, PAM interacting‌; REC1, recognition lobe 1; REC2, recognition lobe 2; REC3 recognition lobe 3; RuvC I, RuvC‐like nuclease domain I; RuvC II, RuvC‐like nuclease domain II; RuvC III, RuvC‐like nuclease domain III; WED, wedge domain.

CRISPR‐Cas systems are classified by effector architecture into Class 1 (types I, III, IV, and VII) using multi‐subunit effector complexes and Class 2 (types II, V, and VI) utilizing single‐protein effectors^15^, ^16^. Notably, interference mechanisms diverge significantly between classes. Class 2 systems use streamlined effectors like *Streptococcus pyogenes* Cas9 (SpyCas9) (Figure 2A), which uses a single nuclease domain guided by crRNA and trans‐activating crRNA (tracrRNA)^17^. In contrast, Class 1 systems show greater complexity, exemplified by the type I‐F Cascade complex (Figure 2B). This crRNA‐guided assembly (Cas5f^1^:Cas6f^1^:Cas7f^6^:Cas8f^1^) scans DNA via Cas8f‐mediated PAM recognition, triggers R‐loop formation, and recruits the Cas2/3 nuclease for targeted degradation (Figure 2C)^2^.

Beyond their natural role as molecular sentinels, CRISPR‐Cas systems have revolutionized genetic engineering. Following Charpentier's discovery of the three‐component system, that is, Cas9, crRNA, and tracrRNA in *S. pyogenes* ^17^, Siksnys et al. demonstrated reprogrammable DNA targeting in 2012^18^. By 2013, Zhang and Church independently established CRISPR‐Cas9 editing in eukaryotic cells^19^, ^20^, catalyzing a paradigm shift in biotechnology. Subsequent innovations span precision tools including base editors^21^, prime editors, and RNA‐targeting effectors^20^, collectively transforming CRISPR‐Cas from a fundamental biological discovery into a versatile technological platform.

## EVOLUTIONARY COUNTERMEASURES OF CRISPR‐CAS: Acr PROTEINS

In the continuing molecular arms race between prokaryotes and MGEs, phages have evolved potent inhibitors known as Acr proteins to neutralize host defenses (Figure 1). First characterized in *Pseudomonas aeruginosa* by Davidson and Bondy‐Denomy in 2013^4^, more than 120 phylogenetically diverse Acr families have since been identified, reflecting their critical role in phage–host coevolution^22^. These inhibitors typically suppress CRISPR‐Cas function through four primary mechanisms: blocking nucleic acid binding, preventing target cleavage, disrupting effector assembly (specific to Class 2 systems), or hydrolyzing cyclic oligoadenylate secondary messengers (in type III systems)^23^. Although most characterized Acr proteins act via high‐affinity steric occlusion of Cas proteins, this canonical inhibition paradigm often requires supra‐stoichiometric inhibitor ratios for complete suppression. The canonical paradigm emerged with early Acr proteins such as AcrIF1 and AcrIF2, which bind the type I‐F Cascade complex stoichiometrically^24^. Structural and mutational analyses revealed two distinct modes of steric occlusion: the backbone‐targeting AcrIF1 and DNA‐recognition disruptor AcrIF2^25^, ^26^. In parallel, a third strategy was discovered in which AcrIF3 sequesters the Cas2/3 nuclease, thereby blocking its recruitment to Cascade and directly inhibiting cleavage activity^27^. Collectively, these high‐affinity steric occlusion mechanisms constitute the canonical inhibition framework for Acr proteins.

Recent studies have also characterized several canonical Acr proteins with intriguing characteristics. Among these, AcrIIA11, AcrIIA15, AcrIIA28, and AcrIIC4 directly interact with Cas9 homologs to exert inhibition. Structurally resolved Acr‐Aca (anti‐CRISPR‐associated) fusion proteins AcrIIA11 and AcrIIA15 show dual functionality. They stably bind Cas9 to suppress CRISPR‐Cas systems while autoregulating their own genetic loci^28^, ^29^. AcrIIA28, another canonical Acr protein, inhibits SpyCas9 by binding its recognition lobe 3 (REC3) domain. Notably, this metalloprotein requires a zinc ion (Zn²⁺) for activity, which is essential for effective Cas9 inhibition^30^. AcrIIC4 occupies the crevice between the REC1 and REC2 domains of *Haemophilus parainfluenzae* Cas9 (HpaCas9). Its extensive interactions restrict REC2 domain mobility, preventing formation of the full‐length guide RNA:target DNA heteroduplex and consequently blocking Cas9 nuclease activation^31^, ^32^. Additionally, AcrIIIA1 binds Csm2 within the Cas10–Csm effector complex, attenuating both Cas10 DNase activity and second messenger production^33^. AcrIIIB2 interacts with the Cmr4α subunit, forming a complex with target RNA and the Cmr‐α ribonucleoprotein to inhibit RNase, single‐stranded DNase (ssDNase), and cyclic oligoadenylate (cOA) synthesis activities^34^. Conversely, AcrVIB1 impedes immunity by binding Cas13b and promoting nonproductive crRNA binding that exposes Cas13b to RNase attack^35^. Although extensively reviewed elsewhere^23^, ^36^, this paradigm provides essential context for understanding recently discovered noncanonical inhibitory strategies, which constitute the focus of this work.

The limitations of canonical Acr proteins highlight the need to identify noncanonical Acr with novel mechanisms, as outlined in our earlier work^8^, ^9^. Meanwhile, analogous to pharmacotherapy requiring precise spatiotemporal control, CRISPR‐Cas applications demand regulated activity. Acr proteins, particularly those with potent, noncanonical mechanisms, provide a natural blueprint for developing such control mechanisms, positioning them as invaluable tools for refining the precision and safety of genome editing technologies.

## NONCANONICAL INHIBITION MECHANISM OF Acr PROTEINS

Ongoing research continues to uncover noncanonical Acr strategies that transcend simple steric blockade. Enzymatic Acr proteins such as AcrIF11^37^, an ADP‐ribosyltransferase, and AcrVA5^38^, an acetyltransferase, covalently modify critical Cas protein residues, permanently disabling target recognition without stable complex formation. AcrIF24 represents another notable example, serving as multifunctional inhibitors that combine CRISPR suppression with transcriptional autoregulation. The C‐terminal helix‐turn‐helix domain (HTH) domain of AcrIF24 represses *acr* operon expression while inducing promiscuous DNA binding by the Csy complex^39^. Similarly, AcrIF5 conformationally locks DNA‐bound Cascade, allosterically destabilizing the complex to prevent Cas3 recruitment^40^. Meanwhile, AcrIIA1 binds with high affinity to the catalytic HNH domain of Cas9, leading to Cas9 degradation specifically *in vivo* ^41^. AcrIIA22, on the other hand, introduces nicks in target DNA, thereby thereby reducing Cas9's affinity for its DNA substrate^42^. Additionally, AcrVA1 inactivates Cas12a by cleaving its cognate crRNA^43^. These mechanisms operate substoichiometrically or catalytically, enhancing their potency and revealing novel evolutionary solutions to CRISPR evasion, and highlighting the continuous investigation on the noncanonical Acr strategies.

### Noncanonical inhibition at the adaptation stage

#### Interference with protospacer acquisition in CRISPR‐Cas systems

Acr proteins predominantly target the interference phase of CRISPR‐Cas systems, but recent studies have identified rare exceptions that disrupt protospacer integration^44^, ^45^, a critical step in immune memory formation. Through systematic screening of 136 putative Acr proteins, Bi et al. identified AcrVA5, an inhibitor via the steric mechanism, as the first characterized inhibitor of spacer acquisition in type I‐C/II‐A systems^44^.

**Figure 3:**
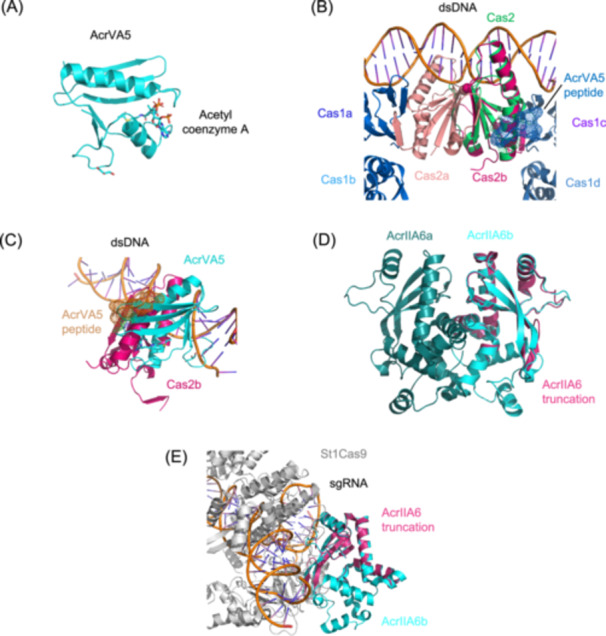
AcrVA5 and a truncated AcrIIA6 inhibit CRISPR immune memory formation through enzymatic modification and steric blockade. (A) Overall structure of AcrVA5 (PDB IDe: 6IUF). (B) Structural alignment between the Cas1–Cas2‐dsDNA complex (PDB ID: 5XVN) and the Cas2–AcrVA5 peptide (PDB ID: 8IA4), revealing how AcrVA5 binding sterically hinders the integrase complex. (C) Structural alignment between full‐length AcrVA5 (PDB ID: 6IUF) and AcrVA5 peptide from the Cas2–AcrVA5 complex (PDB ID: 8IA4). (D) Structural alignment between the AcrIIA6 dimer (PDB ID: 6RJA) and the predicted AcrIIA6 truncation. (E) Structural alignment between the predicted AcrIIA6 truncation and the St1Cas9–sgRNA–AcrIIA6 complex (PDB ID: 6RJA), showing the truncated monomer's interface with Cas9. dsDNA, double‐stranded DNA; St1Cas9, *Streptococcus thermophilus* CRISPR1‐Cas9.

AcrVA5, a 92‐residue protein from *Moraxella bovoculi* prophage, was initially characterized as an acetyltransferase targeting MbCas12a from the type V‐A system (Figure 3A). By acetylating K635, a residue essential for PAM recognition, AcrVA5 abolishes dsDNA binding and cleavage during interference^38^. Subsequent work revealed its secondary activity as acetylation of Cas2, a conserved component of the Cas1–Cas2 integrase complex critical for spacer acquisition^44^. Biochemical analysis demonstrates that AcrVA5 binds Cas2 (*K*_d_ ≈ 1.1 μM) weakly and acetylates K55 at the Cas2–AcrVA5 interface, mirroring its modification of Cas12a^38^. While this modification inactivates Cas2's nonessential endonuclease activity, it negligibly impacts protospacer integration, as host nucleases compensate for DNA processing^46^, ^47^, ^48^. Unexpectedly, AcrVA5 also disrupts integration through steric hindrance. Crystal structure and EMSA data reveal that AcrVA5 binding induces partial disassembly of the Cas1–Cas2–protospacer complex, physically blocking spacer incorporation (Figure 3B,C)^44^. This steric inhibition, rather than acetylation, likely drives its anti‐acquisition phenotype.

In type II‐A systems, AcrIIA6 shows context‐dependent inhibition of interference or spacer acquisition. Full‐length AcrIIA6 (183 aa) functions as an allosteric inhibitor of *Streptococcus thermophilus* Cas9 (St1Cas9), dimerizing via its N‐terminal HTH motif, and occludes the crRNA‐binding surface^48^, ^49^. However, a truncated variant (Δ1–59) lacking the HTH domain, while inactive in interference, selectively blocks spacer acquisition in monomeric form (Figure 3D)^45^.

This monomeric AcrIIA6 truncation features a reduced binding interface incapable of stable Cas9–crRNA engagement (Figure 3E) and a critical I23 substitution (vs. N81 in full length) that destabilizes Cas9–RNA interactions^45^. The mechanistic basis for its anti‐acquisition activity remains unresolved. Proposed models include its weak, transient binding to St1Cas9 or other acquisition machinery components (e.g., Cas1–Cas2) or competitive inhibition of protospacer processing factors.

In summary, the discovery of AcrVA5 and the truncated AcrIIA6 reveals that phages can sabotage the acquisition of new immunological memories, thereby preventing the host from adapting to future infections.

### Noncanonical inhibition at the expression and processing stage

#### Inhibition of the Cas expression through translation‐dependent downregulation

While most characterized Acr proteins function through direct interaction or modification with fully translated Cas effectors, a recent study has uncovered a novel, translation‐dependent mRNA degradation mechanism for CRISPR‐Cas inhibition^51^. The type V‐A inhibitor AcrVA2 represents the first documented example of this strategy in prokaryotic systems, demonstrating a sophisticated approach to thwarting CRISPR immunity by targeting Cas protein biogenesis at the translational level^51^.

This novel mechanism involves AcrVA2 recognition of conserved structural features within the nascent N‐terminal residues Leu‐Ser‐Lys‐Thr (LSKT) polypeptide of Cas12a during translation, which subsequently triggers degradation of the corresponding mRNA before translation is complete^51^. The system demonstrates remarkable specificity, as evidenced by its activity across diverse Cas12a orthologs regardless of promoter or codon usage, and its dependence on intact N‐terminal amino acid sequences rather than nucleotide composition. While the precise degradation machinery remains unknown, this strategy offers evolutionary advantages by targeting essential, conserved domains and preventing Cas12a replenishment.

**Figure 4:**
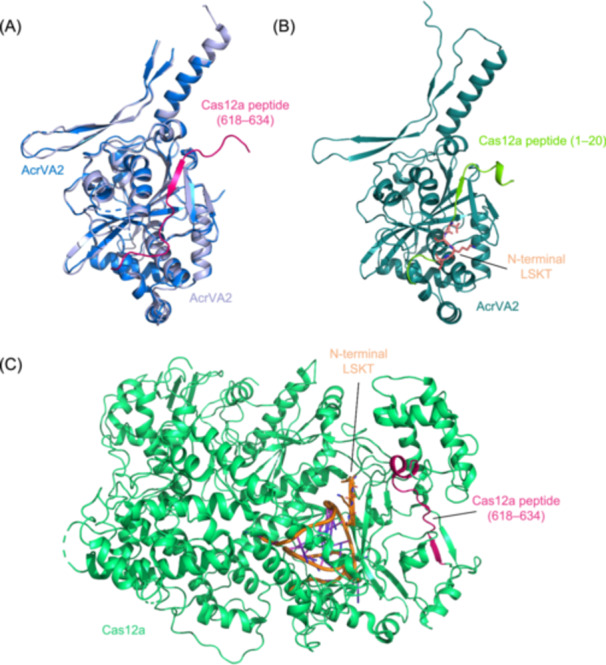
AcrVA2 targets distinct Cas12a regions to inhibit translation. (A) Structural alignment of free AcrVA2 (PDB ID: 7CI1) and the AcrVA2–Cas12a_618__–__634_ peptide complex (PDB ID: 7CI2). (B) Alignment of the AcrVA2–Cas12a_618__–__634_ complex (PDB ID: 7CI2) with the predicted AcrVA2–Cas12a_1__–__20_ complex, indicating a similar binding mode for the N‐terminal LSKT motif (residues Leu‐Ser‐Lys‐Thr, a conserved sequence crucial for AcrVA2 recognition). (C) Location of the N‐terminal LSKT motif and the 618–634 peptide within the MbCas12a–crRNA complex (PDB ID: 6IV6), highlighting the two separate Cas12a regions targeted by AcrVA2. MbCas12a, *Moraxella bovoculi* Cas12a.

Structural biology provides crucial complementary insights. The solved crystal structure of AcrVA2 bound to a Cas12a peptide (residues 618–634) reveals a stable interaction interface distinct from the N‐terminal LSKT motif (Figure 4A,C)^52^, adjacent to the acetylation region targeted by AcrVA5^38^. While biochemically validated, the functional link between this interaction and translation‐dependent degradation requires further investigation. Notably, our structural prediction of AcrVA2 complexed with the Cas12a N‐terminal peptide (residues 1–20, including LSKT) indicates a similar binding mode to the Cas12a_618–634_ peptide (Figure 4B,C), suggesting broader capacity for AcrVA2–Cas12a interactions. Future studies should address the biological significance of the AcrVA2–Cas12a_618–634_ interface.

It is noteworthy that the prophage encoding AcrVA2 often also carries another inhibitor, AcrVA1, implementing a powerful dual inhibition strategy. This combines AcrVA2's pretranslational mechanism, that is, degrading *cas12a* mRNA, with AcrVA1's posttranslational mechanism, that is, cleaving the crRNA within mature Cas12a–crRNA complexes. This two‐pronged attack, preventing new Cas12a synthesis while inactivating existing complexes, confers more effective suppression of CRISPR immunity than either Acr protein could achieve in isolation. Such synergistic strategies, where one Acr protein reduces Cas protein expression and another inactivates pre‐formed complexes, have also been documented in phage‐encoded type II‐A and type I‐F systems^40^, ^41^.

Conceptually, this translation‐coupled regulation bears similarity to eukaryotic quality control systems like tubulin autoregulation^53^, and yet represents the first known example in prokaryotic defense systems. The discovery expands our understanding of phage–host conflicts and opens avenues for developing RNA‐targeting tools and studying translation‐coupled regulation in bacteria. In contrast to classical Acr proteins that inhibit pre‐assembled Cas complexes, AcrVA2 operates at the pre‐translational level by degrading Cas12a mRNA, thereby preventing Cas protein synthesis, a strategy distinct from the canonical post‐translational inhibition paradigm.

#### Mechanistic dissociation and assembly inhibition of the CRISPR‐Cas cascade

Fully assembled CRISPR‐Cas Cascade complexes show exceptional stability, with minimal subunit dissociation/reassociation under physiological conditions. This was demonstrated by the type I‐F Cascade remaining intact after incubation with an 18‐fold molar excess of RNase A at 37°C for 30 min^24^.

**Figure 5:**
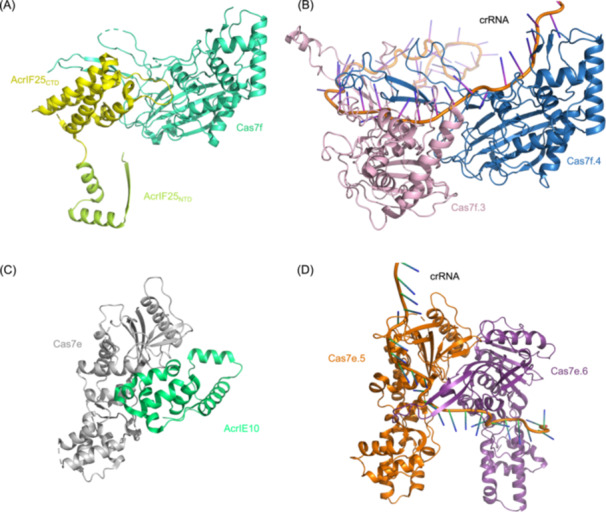
AcrIF25 disassembles and AcrIE10 prevents assembly of the CRISPR Cascade by targeting Cas7 interfaces. (A) Structure of AcrIF25 bound to Cas7f (PDB ID: 8JDI), showing how its C‐terminal domain mimics Cas7–Cas7 interactions. (B) Structure of Cas7f.3 and Cas7f.4, as well as the crRNA, in the type I‐F Cascade (PDB ID: 5B45), illustrating the subunit interface targeted by AcrIF25 for disassembly. (C) Structure of AcrIE10 bound to KpCas7e (PDB ID: 8JI9), demonstrating how it occupies the conserved intermolecular groove. (D) Structure of Cas7e.5 and Cas7e.6, as well as the crRNA, in the type I‐E Cascade (PDB ID: 5F9F), showing the interface blocked by AcrIE10 to prevent complex assembly. CTD, C‐terminal domain; NTD, N‐terminal domain; KpCas7e, *Klebsiella pneumoniae* Cas7e.

AcrIF25 represents a novel mechanistic paradigm. Unlike Acr proteins that block target recognition or prevent complex assembly, AcrIF25 actively disassembles mature type I‐F Cascade complexes^54^. Structural analyses reveal that its C‐terminal domain (CTD) binds Cas7 subunits using an interface mimicking Cas7–Cas7 interactions, sequentially removing subunits from one complex terminus (Figure 5A,B). Remarkably, this energy‐independent disassembly occurs without enzymatic activity or ATP hydrolysis, establishing AcrIF25 as the first known protein capable of dismantling stable macromolecular complexes sans external energy. While multiple Acr proteins, including AcrIF1/2^25^, AcrIF5^40^, AcrIF8/9^55^, AcrIF14^56^, ^57^ and AcrIF24^39^, ^58^, ^59^, target Cas7 subunits, these primarily disrupt PAM recognition or dsDNA loading rather than complex dissociation.

This dissociation mechanism is particularly potent *in vivo*, as the loss of Cas7 protection renders the crRNA highly susceptible to degradation by cellular nucleases, resulting in irreversible CRISPR‐Cas inactivation. Crucially, *in vitro* assays demonstrate that AcrIF25 disrupts pre‐assembled Cascade, which was a significant advantage, as phages must often neutralize CRISPR‐Cas systems that are already active upon infection. Additionally, by binding Cas7 subunits, AcrIF25 may also prevent the *de novo* assembly of Cascade complexes, further enhancing its inhibitory efficiency. Whereas most canonical Acr proteins function by sterically blocking target DNA or nuclease recruitment, AcrIF25 represents a novel paradigm by actively disassembling pre‐formed Cascade complexes without energy input, highlighting a departure from the traditional “masking” or competitive inhibition strategies.

AcrIE10 uses a complementary strategy through assembly inhibition^60^. Structural analysis of the AcrIE10–KpCas7e complex reveals a unique protein fold targeting Cas7e's conserved intermolecular groove (Figure 5C), analogous to AcrIF25^54^. By occupying this interface, AcrIE10 simultaneously blocks Cas7–Cas7 interactions and crRNA loading, preventing surveillance complex formation at initial assembly stages (Figure 5D), distinguishing it from Acr proteins targeting pre‐formed complexes. Phylogenetic analyses show broad conservation across bacterial phyla, indicating strong evolutionary selection for this type I‐E inhibitor.

We propose AcrIE10 may also dissociate pre‐formed Cascade complexes, analogous to AcrIF25 Figure 5. This hypothesis is supported by two lines of evidence. First, structural comparisons reveal parallels in the Cas7‑binding modes of AcrIF25–Cas7f and AcrIE10–Cas7e (Figure 5). Second, overexpression of AcrIE10 severely impedes Cascade assembly. This effect cannot be explained solely by inhibition of assembly, because direct binding to Cas7e is insufficient to fully deplete its subunits. However, experimental validation is required to determine whether AcrIE10 actively disassembles mature complexes.

Collectively, these findings demonstrate that Acr proteins can block CRISPR immunity by targeting the very biogenesis of the effector machinery, using strategies ranging from translation‐coupled mRNA degradation to the active disassembly of pre‐formed surveillance complexes.

#### Molecular mimicry by Cas homologous Acr proteins

**Figure 6:**
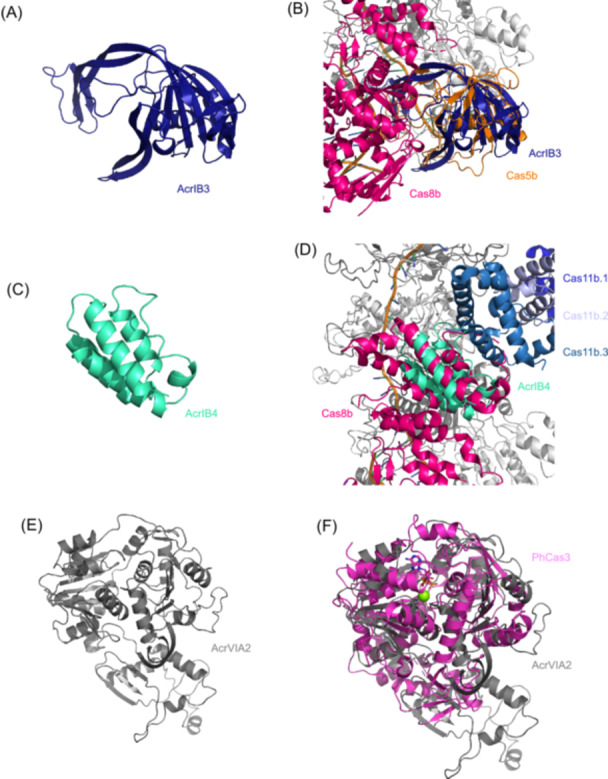
Acr proteins sabotage CRISPR through molecular mimicry. (A, B) Predicted structure of AcrIB3 (A) and its alignment with the type I‐B Cascade (PDB ID: 8H67) (B), showing how it mimics Cas5 to displace the native subunit during assembly. (C, D) Predicted structure of AcrIB4 (C) and its alignment with the type I‐B Cascade (PDB ID: 8H67) (D), illustrating homology to the Cas8b C‐terminal domain. (E, F) Predicted structure of AcrVIA2 (E) and its alignment with *Pyrococcus horikoshii* Cas3 (PhCas3) (PDB ID: 8WTL) (F), revealing its evolutionary origin. Despite this homology, AcrVIA2 unexpectedly targets the type VI‐A Cas13 system.

A recent study revealed that AcrIB3 is a structural mimic of the type I‐B Cascade subunit Cas5, with which it shares 38% sequence identity, and competitively displaces endogenous Cas5 during complex assembly^61^ (Figure 6A). Immunoprecipitation assays confirm its integration into Cascade concomitant with native Cas5 exclusion. AlphaFold 2 predictions highlight critical structural divergences that AcrIB3 lacks Cas5's 18‐residue “hook” domain essential for Cas8b binding but features a unique 6‐residue extended loop predicted to contact Cas7 (Figure 6B). Functional validation shows that restoring the hook domain or deleting this loop abolishes Acr activity. This defective subunit mechanism disrupts target DNA engagement without impeding complex formation, representing a sophisticated interference sabotage strategy. Unlike canonical Acr proteins that typically bind Cas effectors to occlude functional sites, AcrIB3 uses molecular mimicry to replace endogenous Cas5 during assembly, thereby sabotaging complex function from within, a mechanism that diverges from the conventional steric blockade model. Similarly, AcrIB4 shares 38% identity with the C‐terminal domain (CTD) of Cas8b and impairs type I‐B CRISPR‐Cas immunity^61^ (Figure 6C,D). As AcrIB4 does not abolish the assembly of the type I‐B Cascade, its precise mechanism requires further validation.

In contrast, AcrVIA2, despite sharing 24% homology with the Cas3 helicase domain, unexpectedly targets the RNA‐guided type VI‐A system^61^ (Figure 6E,F). It inhibits Cas13 by depleting mature crRNAs without affecting pre‐crRNA transcription or processing. Mutation of conserved DEAD‐box residues (DEFD → AAFD) abolishes activity while preserving protein stability, confirming ATPase dependence. Crucially, AcrVIA2 shows no stable interaction with Cas13 and fails to disassemble pre‐formed Cas13–crRNA complexes *in vitro*. These observations suggest a mechanism involving crRNA degradation during ribonucleoprotein (RNP) biogenesis, potentially via recruitment of cellular nucleases or intrinsic RNase activity.

### Noncanonical inhibition at the interference stage

#### Enzymatic disarming of the Cas surveillance complex CRISPR interference

In addition to stable binding and complex dissociation, a potent and substoichiometric mechanism of CRISPR‐Cas inhibition involves enzymatic inactivation. Enzymatic Acr proteins catalyze posttranslational modifications (PTMs) or nucleolytic cleavage of core Cas components or their nucleic acid substrates, providing a highly efficient and specific strategy for immune evasion. Although less common than stoichiometric inhibitors, enzymatic Acr proteins represent a sophisticated adaptation in the continuing phage–bacteria arms race.

**Figure 7:**
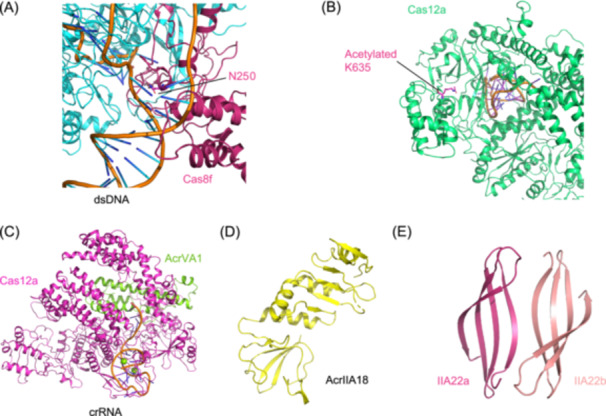
Diverse enzymatic activities of Acr proteins mediate potent and substoichiometric inhibition of CRISPR interference. (A) Cartoon structure of the type I‐F Csy complex (PDB ID: 6NE0), highlighting N250 on the Cas8f subunit. This residue is ADP‐ribosylated by AcrIF11, thereby blocking protospacer adjacent motif (PAM) recognition and DNA binding. (B) Cartoon structure of Cas12a (PDB ID: 6IV6) showing the acetylated K635, a modification introduced by AcrVA5 that abolishes target DNA binding and cleavage. (C) Cartoon structure of the AcrVA1–crRNA–Cas12a complex (PDB ID: 6NMD), illustrating how the RNase activity of AcrVA1 cleaves the crRNA spacer to inactivate the DNA surveillance complex. (D) Cartoon structure of AcrIIA18 (PDB ID: 7VLM), which functions similarly to AcrVA1 by cleaving the sgRNA to inhibit Cas9. (E) Cartoon structure of AcrIIA22 (PDB ID: 7JTA), a topoisomerase‐like Acr that nicks plasmid DNA to reduce Cas9's affinity for its target.

A key example is AcrIF11, a phage‐ and plasmid‐encoded ADP‐ribosyltransferase (ART) that targets the type I‐F Csy complex. As established by Niu et al.^37^ and further elucidated by Chen et al.,^62^ AcrIF11 transfers an ADP‐ribose group from NAD⁺ to N250 of the Cas8f subunit, thereby blocking target DNA binding and preventing CRISPR interference, a mechanism noted for its high specificity and minimal off‐target activity *in vivo* (Figure 7A). Its enzymatic nature allows AcrIF11 to outperform stoichiometric inhibitors under high CRISPR pressure, such as during Cas protein overexpression or multi‐spacer targeting, where its substoichiometric activity enables potent and durable inhibition.

Notably, the repertoire of enzymatic Acr proteins extends well beyond ADP ribosylation, encompassing diverse catalytic activities that sabotage CRISPR interference through distinct pathways. For instance, the aforementioned AcrVA5 uses acetyltransferase activity to covalently modify K635 in Cas12a, abolishing its ability to bind target DNA^38^ (Figure 7B). Meanwhile, AcrVA1 acts as a sequence‐specific RNase that cleaves the crRNA within the Cas12a–crRNA complex, rendering it dysfunctional^43^ (Figure 7C). In a similar RNase‐driven strategy, AcrIIA18 cleaves the sgRNA of Cas9 to a truncated form that fails to activate DNA cleavage, effectively neutralizing the CRISPR‐Cas9 system^63^ (Figure 7D). Moreover, AcrIIA22 introduces nicks into plasmid DNA via its topoisomerase‐like activity, thereby reducing Cas9s affinity for its target substrate^42^ (Figure 7E). Additionally, AcrIII‐1 degrades cyclic oligoadenylate (cOA) second messengers in type III systems through its cyclic nucleotide nucleosidase activity^64^. These findings underscore enzymatic inhibition as a crucial adjunct to canonical steric occlusion and reveal a vast landscape of novel inhibitory mechanisms yet to be fully uncovered in the Acr arsenal.

However, the enzymatic strategy carries inherent vulnerabilities. As shown for AcrIF11, the ADP ribosylation of Cas8f can be removed *in vitro* by macrodomain proteins such as human MacroD2, suggesting that host‐encoded “eraser enzymes” could potentially counteract such Acr proteins^62^. Similarly, the reversibility of AcrVA5‐mediated acetylation underscores a broader theme: while enzymatic Acr proteins provide catalytic efficiency and high specificity, they also risk being neutralized by host defense systems^65^. This stands in contrast to stoichiometric inhibitors, which often engage larger protein interfaces and are less amenable to simple enzymatic reversal.

Thus, the evolutionary choice between enzymatic and stoichiometric inhibition represents a trade‐off between catalytic potency and the potential for host‐mediated counter‐defense, a dynamic that continues to shape the molecular arms race between phage and bacterium. The expanding diversity of catalytic mechanisms used by Acr proteins highlights enzymatic inhibition as a versatile and potent form of interference, promising both new biological insights and innovative tools for controlling CRISPR‐Cas activity.

#### Direct sequestration of R‐loop DNA in CRISPR interference

**Figure 8:**
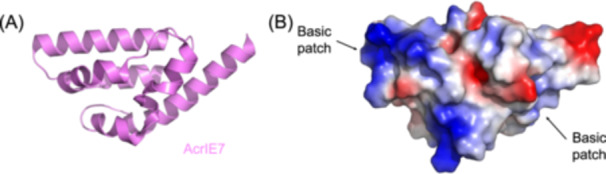
AcrIE7 inhibits CRISPR interference by directly binding and sequestering R‐loop single‐stranded DNA (ssDNA). (A) Cartoon representation of the AcrIE7 monomer, revealing a seven‐helix bundle fold (PDB ID: 9KJB). (B) Electrostatic surface potential of AcrIE7, showing distinct basic (blue) patches. These positively charged regions are critical for the specific recognition and binding of ssDNA. This unique mechanism allows AcrIE7 to occlude the displaced non‐target strand within the R‐loop, thereby inhibiting Cas3‐mediated DNA degradation.

AcrIE7 uses a unique, nucleic acid‐targeting strategy to block CRISPR‐Cas immunity by binding exposed single‐stranded DNA (ssDNA) within the R‐loop structure^66^. Structural and biochemical analyses reveal that AcrIE7 adopts a monomeric seven‐helix bundle fold with two electropositive surface patches (Figure 8A,B), enabling specific ssDNA binding without affinity for double‐stranded DNA (dsDNA). Mechanistically, AcrIE7 inhibits Cas3‐mediated cleavage by sequestering ssDNA substrates. *In vitro*, it prevents Cas3 nuclease activity on free ssDNA through direct occlusion and protects the displaced non‐target strand ssDNA within Cascade‐formed R‐loops, thereby blocking DNA degradation in type I‐E/I‐C systems.

This mechanism fundamentally diverges from canonical Acr paradigms. Unlike Acr proteins targeting protein components (e.g., AcrIE1 blocking Cas3 recruitment or AcrIE10 disrupting Cascade assembly), AcrIE7 directly hijacks the nucleic acid intermediate of CRISPR interference. Although AcrIE7 shows basal ssDNA affinity, its inhibitory efficacy is enhanced within the context of Cascade complexes, suggesting potential synergy with Cascade‐induced DNA unwinding during R‐loop formation. However, the molecular basis for its selectivity toward the type I‐E system remains to be fully clarified, requiring future elucidation of the Cascade–R‐loop–ssDNA–AcrIE7 complex.

In summary, noncanonical inhibition at the interference stage is characterized by strategies that go beyond simple steric blocking, including the direct sequestration of nucleic acid intermediates, enzymatic inactivation of Cas effectors, and molecular mimicry to sabotage complex function.

## CONCLUDING REMARKS

The ongoing phage–host arms race remains a fertile ground for biological discovery. The Acr field has advanced rapidly since our last review, with over 100 new publications underscoring the growing mechanistic diversity and significance of Acr. We anticipate continued expansion in the number of identified Acr proteins, revealing increasingly sophisticated inhibition strategies. Notably, recent work has extended Acr activity beyond canonical interference stages. To provide a clear overview of the diverse Acr proteins discussed in this review, their targets, and their noncanonical mechanisms, we summarize key examples in Table 1.

**Table 1:** Summary of representative noncanonical anti‐CRISPR (Acr) proteins, their targets, and inhibition mechanisms.

Family	Antitype	Molecular weight (kDa)*	Structure (PDB ID)	Targeting	Binding subunit	Known inhibition mode
AcrIF11	I‐F	14.52	6KYF, 8DWQ	Cascade	Cas8f, Cas7f	ADP‐ribosylation of N250 of Cas8f and blockage of DNA binding
AcrIF25	I‐F	18.18	8JDI, 8JDH	Cascade	Cas7f	Active disassembly of pre‐formed Cascade by mimicking Cas7–Cas7 interactions
AcrIE7	I‐E	11.94	9KJB, 9LO1, 9LO2, 9L2Q	R‐loop ssDNA	ssDNA (non‐target strand)	Binding and sequestration of ssDNA in R‐loop, followed by blockage of Cas3 cleavage
AcrIE10	I‐E	13.21	8JI9	Cascade	Cas7e	Prevention of Cascade assembly via blockage of Cas7–Cas7 interactions and crRNA loading
AcrIB3	I‐B	27.24	N/A	Cascade	N/A	Mimicry of Cas5 and competitive displacement of native Cas5 during assembly
AcrIB4	I‐B	9.1	N/A	Cascade	N/A	Homology to Cas8b C‐terminal domain and impairment of immunity (mechanism unclear)
AcrIIA6	II‐A	21.44 (full)/14.60 (trunc)	6EYX, 6EYY, 6RJ9, 6RJG, 6RJA, 6RJD	Cas9	REC lobe	Allosteric inhibition of St1Cas9 and blockage of spacer acquisition by truncated form
AcrIIA18	II‐A	20.89	7VLM	Cas9‐sgRNA	sgRNA	Cleavage of sgRNA and prevention of Cas9 activation
AcrIIA22	II‐A	5.97	7JTA	Plasmid DNA	DNA	Nicking of plasmid DNA and reduction of Cas9 affinity for target
AcrVA1	V‐A	20.24	6NMD, 6NMA, 6NMC	Cas12a‐crRNA	crRNA	Cleavage of crRNA within Cas12a‐crRNA complex by RNase activity
AcrVA2	V‐A	36.74	7CI1, 7CI2	Cas12a mRNA	Nascent Cas12a peptide	Triggering of translation‐coupled mRNA degradation by recognition of LSKT motif
AcrVA5	V‐A	10.57	6IUF, 8IA4, 6IV6	Cas12a/Cas2	Cas12a (K635), Cas2 (K55)	Acetylation of Cas12a (K635) and Cas2 (K55) as acetyltransferase; also steric blockage of integration
AcrVIA2	VI‐A	61.09	7XMW	Cas13‐crRNA	crRNA (indirect)	Depletion of mature crRNAs via ATPase‐dependent mechanism

crRNA, CRISPR RNA; LSKT, residues Leu‐Ser‐Lys‐Thr; N/A, not available; sgRNA, single‐guide RNA; ssDNA, single‐stranded DNA.

For example, AcrVA5 disrupts spacer acquisition by acetylating Cas2 and promoting the disassembly of integrase complexes^44^, whereas a truncated form of AcrIIA6 impedes protospacer integration^50^. Intriguingly, the evolutionary pressure for phages to encode acquisition‐blocking Acr proteins remains debated, given the low natural spacer integration frequency (~10⁻⁷ per cell) and potential trade‐offs between immunity and autoimmunity^67^. AcrVA5's modest affinity for Cas2 (with a *Kd* much higher than that of the Cas1–Cas2 interaction) and subtype specificity suggest that its acquisition‐blocking role may be incidental rather than selected^68^. Furthermore, from an evolutionary perspective, the inhibition of spacer acquisition by proteins like AcrVA5 may provide a crucial “pre‐emptive” advantage for phages confronting hosts with highly efficient adaptation capabilities, preventing the establishment of targeted immune memory despite the low natural frequency of spacer integration. Regardless of their evolutionary origin, such inhibitors offer valuable tools for probing spacer integration mechanics and refining off‐target control in CRISPR applications.

At the expression and processing stage, Acr proteins increasingly reveal novel mechanisms for sabotaging CRISPR complex assembly. For instance, AcrID1 and AcrIE10 bind Cas10 and Cas7, respectively, preventing Cascade assembly and crRNA loading^60^, ^69^, ^70^. Strikingly, AcrIF25 can disassemble fully formed Cascade complexes, previously considered highly stable, by extracting terminal Cas7 subunits^54^. This ATP‐independent disassembly mechanism, contrasting with AcrIE10's preassembly blockade, highlights a potent inhibitory strategy. Given that many macromolecular complexes rely on repeating subunits, AcrIF25's principles may inspire engineered disassembly factors for diverse systems. Nevertheless, AcrVA2 further exemplifies preinterference inhibition by degrading *cas12a* mRNA, thereby establishing a new paradigm for the potential regulation of CRISPR‑Cas genome editing^51^.

Transcriptional crosstalk between Acr proteins and CRISPR‐Cas systems adds further complexity. SpyCas9 auto‐represses its expression, but phage Acr proteins relieve this repression, triggering rapid CRISPR‐Cas upregulation during infection^71^. Similarly, cas‐regulating RNAs (CreRs) mediate autorepression in type I‐B, I‐E, and V‐A systems via partial promoter complementarity^72^. Conversely, some phages exploit CRISPR‐Cas effectors to repress toxin genes such as (CreTA/CrePA) modules; when Acr proteins inhibit the effector, toxin expression halts cell division^73^, ^74^. These findings extend beyond the simple phage–host arms race paradigm, revealing how Acr and CRISPR‐Cas systems are integrated into broader cellular regulatory networks, influencing fundamental processes such as toxin–antitoxin systems and cell division.

The evolutionary dynamics of the arms race are evident in how Acr proteins arise via functional exaptation. For instance, phages repurpose cas genes through imprecise prophage excision, evolving inhibitory functions via domain loss (AcrIB3's “hook”), neomorphic loops, or catalytic repurposing (AcrVIA2's DEAD‐box domain)^61^. This evolutionary path of “molecular repurposing” explains the remarkable efficiency and diversity of Acr proteins: they arise from minor yet functionally transformative tweaks to existing protein scaffolds, rather than evolving entirely new folds from scratch. This mechanism also enables phages to rapidly generate new Acr variants to counter the ever‐changing defensive pressures of their hosts. Structural prediction tools like AlphaFold accelerate Acr discovery and origin tracing^75^, implicating toxins–antitoxins and SOS repair components as potential Acr precursors^76^. However, conflicting reports on Acr activities (e.g., AcrVIA1‐7, AcrIII‐1, and AcrIIA7) emphasize the need for validation in native contexts and caution in extrapolating heterologous data^77^, ^78^, ^79^.

Biotechnologically, Acr proteins provide unparalleled templates for precision control in CRISPR applications. Their natural inhibitory ingenuity transforms CRISPR‐Cas from a constitutive scalpel into a regulatable tool: AcrVA2 enables temporal control of gene editing via translation suppression, AcrIF25 acts as a “reset switch” by disassembling Cascade, and AcrVIA2.1 broadly inhibits DNA/RNA‐editing tools. Future structural studies (e.g., cryo‐EM of AcrIB3‐bound Cascade) will refine mechanistic understanding and guide engineering. Key challenges include elucidating unresolved mechanisms (e.g., AcrVA2's coordination of peptide recognition/mRNA decay), overcoming delivery bottlenecks for *in vivo* use, and assessing ecological risks (e.g., Acr‐driven antibiotic resistance spread). Prioritizing cryo‐ET in cellular contexts, high‐throughput screening for Acr–small molecule hybrids, and metagenomic mining in extreme environments will advance this vibrant field, bridging fundamental virology and safer CRISPR technologies.
